# A cross-sectional study on pelvic floor symptoms in women living with Female Genital Mutilation/Cutting

**DOI:** 10.1186/s12978-021-01097-9

**Published:** 2021-02-12

**Authors:** Alzbeta Binkova, Marion Uebelhart, Patrick Dällenbach, Michel Boulvain, Angèle Gayet-Ageron, Jasmine Abdulcadir

**Affiliations:** 1 Faculty of Medicine, University of Geneva, Rue Michel-Servet 1, 1211 Geneva, Switzerland; 2 Division of Gynaecology, Department of Paediatrics, Gynaecology and Obstetrics, Geneva University Hospitals, Boulevard de la Cluse 30, 1211 Geneva, Switzerland; 3 Clinical Research Center & Division of Clinical Epidemiology, Department of Health and Community Medicine, University of Geneva & Geneva University Hospitals, Geneva, Switzerland

**Keywords:** Female genital mutilation/cutting (FGM/C), Infibulation, Past violent events, Urogynaecological and psychosexual complications, Pelvic floor symptoms, PFDI-20, PFIQ-7

## Abstract

**Background:**

Female Genital Mutilation/Cutting (FGM/C) concerns over 200 million women and girls worldwide and is associated with obstetric trauma and long-term urogynaecological and psychosexual complications that are often under-investigated and undertreated. The aim of this study was to assess the pelvic floor distress and the impact of pelvic floor and psychosexual symptoms among migrant women with different types of FGM/C.

**Methods:**

This cross-sectional study was conducted between April 2016 and January 2019 at the Division of Gynaecology of the Geneva University Hospitals. The participants were interviewed on socio-demographic and background information, underwent a systematic gynaecological examination to assess the presence and type of FGM/C and eventual Pelvic Organ Prolapse (POP), and completed six validated questionnaires on pelvic floor and psychosexual symptoms (PFDI-20 and PFIQ7 on pelvic floor distress and impact, FISI and WCS on faecal incontinence and constipation, PISQ-IR and FGSIS on sexual function and genital self-image). The participants’ scores were compared with scores of uncut women available from the literature. The association between selected variables and higher scores for distress and impact of pelvic floor symptoms was assessed using univariate and multivariable linear regression models.

**Results:**

124 women with a mean age of 31.5 (± 7.5), mostly with a normal BMI, and with no significant POP were included. PFDI-20 and PFIQ-7 mean (± SD) scores were of 49.5 (± 52.0) and 40.7 (± 53.6) respectively. In comparison with the available literature, the participants’ scores were lower than those of uncut women with pelvic floor dysfunction but higher than those of uncut women without such disorders. Past violent events other than FGM/C and forced or arranged marriage, age at FGM/C of more than 10, a period of staying in Switzerland of less than 6 months, and nulliparity were significantly associated with higher scores for distress and impact of pelvic floor symptoms, independently of known risk factors such as age, weight, ongoing pregnancy and history of episiotomy.

**Conclusions:**

Women with various types of FGM/C, without POP, can suffer from pelvic floor symptoms responsible for distress and impact on their daily life.

*Trial registration*. The study protocol was approved by the Swiss Ethics Committee on research involving humans (protocol n°15-224).

## Plain English summary

Female Genital Mutilation/Cutting (FGM/C) involves any injury to the female genital organs for non-medical reasons and concerns over 200 million women and girls worldwide. It is associated with obstetric trauma and long-term pelvic floor and psychosexual complications that are often under-investigated and undertreated. The main objective of this study was to assess the pelvic floor distress and impact of symptoms among migrant women with different types of FGM/C using validated questionnaires available from the literature (PFDI-20 and PFIQ7 on pelvic floor distress and impact, FISI and WCS on faecal incontinence and constipation, PISQ-IR and FGSIS on sexual function and genital self-image). We compared our participants’ scores with scores from the available literature. We assessed the association between known risk factors, FGM/C and higher scores for pelvic floor symptoms’ distress and impact. Our results showed that women with various types of FGM/C can suffer from pelvic floor symptoms responsible for distress and impact on their daily life, independently of known risk factors such as age, weight, ongoing pregnancy or history of episiotomy. Past violent events other than FGM/C and forced or arranged marriage, age at FGM/C of more than 10, a period of staying in Switzerland of less than 6 months, and nulliparity were significantly associated with higher scores for distress and impact of pelvic floor symptoms. We believe that our paper can improve healthcare professionals’ knowledge in screening, recognizing, recording and treating urogynaecological, colorectal-anal, and psychosexual disorders in women living with FGM/C.

## Background

Female Genital Mutilation/Cutting (FMG/C) involves any injury to the female genital organs for non-medical reasons [1]. FGM/C is classified into four types: the cutting of the clitoral hood with or without the clitoral glans (type I); the excision of the labia with or without the clitoral glans (type II); the narrowing of the vaginal orifice by apposition of the labia, with or without excision of the labia and/or the clitoral glans (type III, infibulation); and all other harmful procedures such as pricking, piercing, or cauterization (type IV) [2].

FGM/C is practiced in more than 30 countries and concerns over 200 million women and girls [3], with a prevalence from 1% in Uganda to 98% in Somalia [4]. It is mainly carried out before the age of 15 [3] as a rite of passage to adulthood and marriage [5] and believed to ensure premarital virginity, marital fidelity and clean, pure and beautiful genitals [6]. There is great variability in the types of FGM/C and associated beliefs within and between countries [3]. With migration, FGM/C also exists in high income countries, with over 500,000 women and girls both in Europe and the U.S. [7, 8], and 22,400 in Switzerland [9].

FGM/C can cause long term urogynaecological, psychosexual and obstetrical complications [1, 10, 11]. However, the available literature on such complications mostly consists in retrospective studies, reviews or surveys, without a systematic gynaecological examination of the participants and often without validated questionnaires [12]. Urogynaecological complications are mostly associated with infibulation, which creates an obstacle to urinary and blood flow, causing recurrent urogenital tract infections, dysuria, overactive bladder with urinary incontinence, obstructed micturition, dysmenorrhea, and infertility [1, 11, 13, 14]. Psychosexual complications include spontaneous or provoked vulvar pain, genito-pelvic pain or penetration disorder, depression, anxiety and post-traumatic stress disorder (PTSD) [1, 15]. Negative obstetric outcomes involve increased risks of assisted delivery, episiotomy, severe perineal tears and post-partum haemorrhage [1, 10, 16]. Healthcare professionals often lack knowledge and experience to screen and manage FGM/C complications [17]. In addition, women and girls with FGM/C might not spontaneously mention their cutting or symptoms because of shame or fear [18], or because they are unaware of a potential link between FGM/C and their symptoms [18–20].

The main objective of our study was to investigate the impact and distress of pelvic floor and psychosexual symptoms in a population of women with different types of FGM/C using validated questionnaires available from the literature. Our secondary objectives were: to compare the participants’ scores with the scores reported in the literature among uncut women both with and without pelvic floor dysfunction (PFD); to assess the association between possible risk factors and higher scores for distress and impact of pelvic floor symptoms in women with FGM/C.

## Methods

This cross-sectional study was conducted between April 1, 2016 and January 31, 2019 at the FGM/C outpatient clinic of the Geneva University Hospitals. We included all women with FGM/C who were at least 18, spoke French, English or were accompanied by a certified female interpreter, were not taking hormonal therapy, and did not have a history of hysterectomy.

The study protocol was approved by the Swiss Ethics Committee on research involving humans (protocol n°15-224).

The participants were recruited at the clinic, a few through snowball sampling. Women taking part to the study were encouraged to tell friends about the study and provide our contact details in order for us to be contacted by those who were interested in participating. Prior to the interview, the participants received full information about the study, signed an informed consent, and underwent a gynaecological examination to assess the presence and type of FGM/C as well as eventual Pelvic Organ Prolapse (POP). The gynecological examination was performed before administering the questionnaires to assess the presence of FGM/C. Some eligible women might have been unsure about their history of cutting, for instance if they had undergone FGM/C as a newborn and had no memories of it.

Data collection instruments were administered by three of the investigators (JA, MU, AB) in French, English or with the assistance of a certified female interpreter. The certified interpreters translating the questionnaires were 9, including Somali (1), Arab (1), Amharic (2), Tigrinya (4) and Bambara (1). Our interpreters translate at our clinic in about 30% of the consultations, are routinely involved in research projects among our patients and are trained in the topic of FGM/C. For the three questionnaires validated in English only, the three investigators administered the questionnaires translating English into French consistently.

The investigators administered a questionnaire about sociodemographic and background information and six questionnaires on pelvic floor and psychosexual symptoms and disorders. Since no questionnaires exist for assessing pelvic floor symptoms among women with FGM/C, we used the following existing questionnaires validated among uncut women with and without PFD:

1. Pelvic Floor Distress Inventory short-form (PFDI-20) [21, 22], validated in English and French, assesses the distress of pelvic floor symptoms. It has 46 questions separated into 3 scales: Urinary Distress Inventory (UDI-6), Pelvic Organ Prolapse Distress Inventory (POPDI-8), and ColoRectal-Anal Distress Inventory (CRADI-6). Each scale is scored from 0 to 100, the total score ranges from 0 to 300. Higher scores indicate greater distress of pelvic floor symptoms.
2. Pelvic Floor Impact Questionnaire short-form (PFIQ-7) [21, 22], validated in English and French, estimates the impact of pelvic floor symptoms. It has 31 questions separated into 3 scales: Urinary Impact Questionnaire (UIQ-7), Pelvic Organ Prolapse Impact Questionnaire (POPIQ-7), and ColoRectal-Anal Impact Questionnaire (CRAIQ-7). Each scale is scored from 0 to 100, the total score ranges from 0 to 300. Higher scores indicate greater impact of pelvic floor symptoms on daily life activities.
3. Pelvic organ prolapse Incontinence Sexual Questionnaire—IUGA Revised (PISQ-IR) [23–26], validated in English and French, assesses sexual function and quality in sexually active and inactive women. Sexually Active (SA) questionnaire has 15 questions separated into 6 scales: Arousal and Orgasm (AO), Partner-Related impact on activity (PR), Condition-Specific impact on activity (CS), Condition-specific Impact on quality (CI), Desire (D), and Global Quality (GQ). Non-Sexually Active (NSA) questionnaire has 6 questions separated into 4 scales: Condition-Specific impact on inactivity (CS), Partner-Related impact on inactivity (PR), Condition-specific Impact on quality (CI), and Global Quality (GQ). The scores for each scale are calculated through a Microsoft Excel file provided by the International UroGynaecological Association (IUGA). Higher scores indicate better sexual function.
4. Fecal Incontinence Severity Index (FISI) [27], validated in English, quantifies four types of leakage (gas, mucus, liquid stool, and solid stool) depending on frequency. The total score ranges from 0 to 61. Higher scores indicate greater faecal incontinence.
5. Wexner’s constipation scoring system (WCS) [28], validated in English, assesses constipation severity. It has 8 questions scored from 0 to 4. The total score ranges from 0 to 30. Higher scores indicate greater constipation.
6. Female Genital Self-Image Scale (FGSIS) [29, 30], validated in English, assesses the women’s genital self-image. It has 7 questions scored from 1 to 4. The total score ranges from 7 to 28. Higher scores indicate better genital self-image.

With a total sample size of 124 participants, we were able to describe the questionnaires’ mean scores with a standardized width of the 95% confidence interval of 0.35 [31].

The data were manually entered in an EpiData (software version 4.4.3.1) file and double-checked by two investigators (AB, MU). Statistical analysis was performed using Stata SE (software version 15.0).

We described sociodemographic and background information as numbers and percentages, the questionnaires’ scores as means with standard deviation. We reported our participants’ scores together with those of uncut women both with and without PFD (urinary incontinence and/or POP and/or faecal incontinence) found in the available literature [21, 23, 28, 30, 32–34].

We compared scores and sub-scores of women with FGM/C type III and those of women with FGM/C types I and II. We also compared non-infibulated (FGM/C types I, II, III previously defibulated) and infibulated (FGM/C type III with no previous defibulation or with previous defibulation and reinfibulation) women. Due to small numbers, we used non-parametric Kruskal–Wallis tests.

We explored the association between selected variables and higher scores for pelvic floor symptoms’ distress and impact using univariate and multivariable linear regression models. Variables were selected based on their clinical and literature relevance as risk factors for pelvic floor symptoms. For multivariable models, a backward stepwise method was used to select the variables. Our method of selection was based on our objective to identify risk factors for pelvic floor symptoms in women with FGM/C. Our hypothesis was that type of FGM/C, age at FGM/C, and past violent events other than FGM/C and forced or arranged marriage, could be significantly associated with more severe urogyneocological, colorectal-anal and psychosexual symptoms among the participants, independently of known risk factors such as age, weight, parity, ongoing pregnancy, and history of episiotomy. We tested the association between potentially collinear variables, such as type of FGM/C and age at FGM/C, then verified that the addition of one did not change the association between the other and the outcome scores and kept both in multivariable models. We verified the assumption that residuals were normally distributed (visual inspection). We reported the estimated marginal mean of the different outcome scores with their 95% confidence intervals (95% CI) for each independent variable and the associated p-value for each category of the variables compared to the reference category. Significance was set at a p-value < 0.05.

## Results

One hundred forty-five women accepted to participate. Twenty-one were excluded as they did not return the questionnaires, refused to undergo gynaecological examination, or had exclusion criteria. One woman with FGM/C type IV, a pricking of clitoral hood without visible scar, was also excluded. A total of 124 women were included.

The participants were averagely 31.5 ± 7.5 and had a mostly normal or overweight Body Mass Index (BMI), with a mean of 24.55 ± 5.37 kg/m2. Most had FGM/C type III (n = 74, 59.7%) or type II (n = 41, 33.1%). POP was present in only 2 women (1.6%) and was non-significant (POP-Q stage ≤ 2, above the hymen) (Table 1, Additional file 1: Tables 1 and 2).

**Table 1:** Sociodemographic and background information

Variable	n (%)
Age (years)	31.5 ± 7.5 (range = 19–54)
BMI	24.59 ± 5.37 (range = 15.2–39.0)
< 18.5 (underweight)	10 (8.0)
18.5–24.9 (normal weight)	68 (54.8)
25.0–29.9 (overweight)	23 (18.6)
≥ 30.0 (obesity)	23 (18.6)
Time spent in Switzerland	6.4 ± 7.4 years (range = 13 days–30 years)
< 1 year	28 (22.5)
1–2 years	18 (14.5)
2–3 years	11 (8.9)
3–4 years	10 (8.1)
4–5 years	10 (8.1)
> 5 years	47 (37.9)
Pregnancy at the time of the study	43 (34.7)
1st trimester	4 (9.3)
2nd trimester	23 (53.5)
3rd trimester	16 (37.2)
Type of FGM/C	
Type I	9 (7.2)
Type II	41 (33.1)
Type III, defibulated	40 (32.3)
Type III, infibulated	34 (27.4)
Age at FGM/C	
< 1 year old	35 (28.2)
1–5 years old	16 (12.9)
6–10 years old	40 (32.3)
> 10 years old	11 (8.9)
Unknown	22 (17.7)
Previous defibulation (n_tot_ = 74)	
Yes, in labour	23 (31.1)
Yes, during pregnancy	5 (6.8)
Yes, outside pregnancy	13 (17.6)
No	32 (43.2)
Unknown	1 (1.3)
Previous reinfibulation (n_tot_ = 42)	
No	35 (83.3)
Yes	6 (14.3)
Unknown	1 (2.4)
Past violent events	
Physical	5 (4.0)
Psychological	10 (8.1)
Sexual	4 (3.2)
Several (psychological/physical/sexual)	37 (29.9)
None	64 (51.6)
No answer	4 (3.2)
Delivery (n_tot_ = 78)	
Vaginal	58 (74.4)
Cesarean section	20 (25.6)
Pelvic organ prolapse^4^	
No	122 (98.4)
Yes	2 (1.6)

^1^Acute complications: hemorrhage, pain, urinary pain, infection

^2^Chronic complications: menstrual, sexual, urinary, scarring, obstetric

^3^Other ethnic origin: Burkina Faso, Chad, Djibouti, Gambia, Guinea, Guinea-Bissau, Ivory Coast, Mali, Nigeria, Senegal, Sierra Leone, Sudan, Togo

^4^Prolapsus stage POP- + : 1 (n = 1), 2 (n = 1)

Our study population showed a PFDI-20 mean score of 49.5 ± 52.0 and a PFIQ-7 mean score of 40.7 ± 53.6. In comparison with the literature, our scores and sub-scores were lower than those of women with PFD (PFDI-20 > 90, PFIQ-7 > 60) but higher than those of women without PFD (PFDI-20 < 30, PFIQ-7 < 15) of previous studies in Dutch and South African populations (Table 2) [21, 32, 33].

**Table 2:** PFDI-20^1^ and PFIQ-7^5^ mean scores of our sample and from the literature [21, 32, 33]

	Mean scores ± SD of women FGM/C				Mean scores ± SD of women without FGM/C					
	All (n = 124)	I (n = 9)	II (n = 41)	III (n = 74)	Without PFD^9^			With PFD^9^		
					Dutch	Africaans	Sesotho	Dutch	Africaans	Sesotho
Age	31.5 ± 7.5				47.0 ± 15.3	48.8 ± 13.4	46.5 ± 11.8	58.6 ± 12.2	59.9 ± 9.8	56.4 ± 11.8
Parity	1.3 ± 1.4				NA	3 (1–5)	3 (1–7)	2.3 ± 1.2	3 (0–6)	4 (0–8)
PFDI-20^1^	49.5 ± 52.0	33.8 ± 33.1	45.5 ± 45.4	53.7 ± 57.0	27.1 ± 31.7	16.1 ± 22.3	27.1 ± 31.7	93.4 ± 41.5	131.8 ± 59.2	124.9 ± 55.2
UDI-6^2^	19.6 ± 23.5	14.8 ± 22.0	17.5 ± 20.3	21.3 ± 25.3	12.3 ± 16.1	4.8 ± 6.9	12.3 ± 16.1	44.7 ± 20.7	45.2 ± 27.8	26.0 ± 24.9
POPDI-6^3^	12.2 ± 18.8	5.5 ± 9.3	11.0 ± 17.1	13.7 ± 20.4	5.6 ± 9.8	6.8 ± 12.7	5.6 ± 9.8	22.2 ± 16.5	52.1 ± 23.2	54.7 ± 21.8
CRADI-8^4^	17.8 ± 18.9	14.6 ± 16.8	17.0 ± 19.1	18.6 ± 19.3	9.3 ± 12.4	4.4 ± 6.9	9.3 ± 12.4	26.7 ± 19.5	32.2 ± 25.2	31.7 ± 21.8
PFIQ-7^5^	40.7 ± 53.6	29.1 ± 51.9	32.6 ± 48.1	46.6 ± 56.5	12.7 ± 27.0	13.2 ± 15.1	12.7 ± 27.0	69.0 ± 52.5	115.5 ± 61.6	116.1 ± 62.4
UIQ-7^6^	8.7 ± 18.4	5.3 ± 15.9	7.0 ± 18.7	10.0 ± 18.6	5.0 ± 11.3	4.7 ± 5.8	5.0 ± 11.3	35.1 ± 26.3	44.2 ± 29.1	39.8 ± 27.8
POPIQ-7^7^	16.6 ± 29.4	13.9 ± 33.2	11.3 ± 20.7	20.0 ± 32.7	3.4 ± 10.0	5.1 ± 7.9	3.4 ± 10.0	14.6 ± 22.4	45.4 ± 27.0	46.1 ± 25.6
CRAIQ-7^8^	8.7 ± 19.7	9.5 ± 20.2	7.3 ± 18.4	9.4 ± 20.6	4.3 ± 11.9	2.9 ± 4.9	4.3 ± 11.9	20.9 ± 27.2	25.9 ± 26.2	30.2 ± 26.5

^1^Pelvic Floor Distress Inventory

^2^Urinary Distress Inventory

^3^Pelvic Organ Prolapse Distress Inventory

^4^ColoRectal-Anal Distress Inventory

^5^Pelvic Floor Impact Questionnaire

^6^Urinary Impact Questionnaire

^7^Pelvic Organ Prolapse Impact Questionnaire

^8^ColoRectal-Anal Impact Questionnaire

^9^Pelvic floor dysfunction (urinary incontinence and/or pelvic organ prolapse and/or fecal incontinence)

Our study population showed PISQ-IR scores similar to those of American and British uncut women with PFD, though non-sexually active participants (n = 37, 30.1%) had lower mean CS (Condition Specific impact on activity) and CI (Condition-specific Impact on quality) sub-scores than uncut non-sexually active women with PFD (Table 3) [23]. The FGSIS mean score was 20.9 ± 5.7, similar to the score of a healthy American population (21.3 ± 4.3) (Table 4) [30].

**Table 3:** PISQ-IR 1 mean scores of our sample and from the literature[23]

	Mean scores ± SD of women with FGM/C				Mean scores ± SD of women without FGM/C
	All (n = 124)	I (n = 9)	II (n = 41)	III (n = 74)	With PFD^2^
PISQ-IR^1^					
SA-AO^1^	3.3 ± 1.1 (n = 85)	3.2 ± 0.9 (n = 8)	3.5 ± 0.9 (n = 27)	3.3 ± 1.2 (n = 50)	3.4 ± 0.8
SA-PR^1^	3.4 ± 0.6 (n = 84)	3.8 ± 0.3 (n = 8)	3.4 ± 0.7 (n = 27)	3.4 ± 0.7 (n = 49)	3.2 ± 0.7
SA-CS^1^	4.5 ± 0.8 (n = 84)	4.8 ± 0.4 (n = 8)	4.5 ± 1.1 (n = 27)	4.5 ± 0.7 (n = 49)	4.4 ± 0.7
SA-CI^1^	3.8 ± 0.5 (n = 82)	3.9 ± 0.2 (n = 8)	3.7 ± 0.6 (n = 26)	3.8 ± 0.5 (n = 48)	3.0 ± 0.9
SA-D^1^	2.9 ± 1.0 (n = 83)	2.8 ± 1.2 (n = 8)	3.0 ± 1.0 (n = 26)	2.9 ± 1.0 (n = 49)	3.0 ± 0.9
SA-GQ^1^	3.9 ± 1.1 (n = 80)	3.5 ± 1.3 (n = 8)	3.7 ± 1.2 (n = 26)	4.1 ± 1.0 (n = 46)	3.0 ± 1.1
NSA-CS^1^	1.5 ± 0.7 (n = 37)	1.0 ± 0.0 (n = 1)	1.4 ± 0.5 (n = 13)	1.5 ± 0.8 (n = 23)	2.8 ± 1.0
NSA-PR^1^	2.6 ± 0.8 (n = 35)	2.5 ± 0.0 (n = 1)	2.6 ± 1.0 (n = 11)	2.6 ± 0.8 (n = 23)	2.2 ± 1.0
NSA-CI^1^	1.2 ± 0.6 (n = 36)	1.0 ± 0.0 (n = 1)	1.1 ± 0.3 (n = 13)	1.3 ± 0.7 (n = 22)	2.1 ± 0.1
NSA-GQ^1^	2.3 ± 1.2 (n = 37)	1.5 ± 0.0 (n = 1)	2.0 ± 1.2 (n = 13)	2.6 ± 1.2 (n = 23)	2.9 ± 1.2

^1^Pelvic organ prolapse Incontinence Sexual Questionnaire—IUGA Revised (PISQ-IR) for Sexually Active (SA) and Non-Sexually Active (NSA), Arousal and Orgasm (AO), Partner-Related impact on activity/inactivity (PR), Condition-Specific impact on activity/inactivity (CS), Condition-specific Impact on quality (CI), Desire (D), and Global Quality (GQ)

^2^Pelvic floor dysfunction (urinary incontinence and/or pelvic organ prolapse and/or fecal incontinence)

**Table 4:** FGSIS^1^ mean scores of our sample and from the literature [30]

	Mean scores ± SD of women with FGM/C				Mean scores ± SD of women without FGM/C
	All (n = 124)	I (n = 9)	II (n = 41)	III (n = 74)	With PFD^2^
FGSIS^1^	20.9 ± 5.7 (n = 114)	21.0 ± 6.0 (n = 9)	20.0 ± 6.0 (n = 37)	21.0 ± 5.0 (n = 68)	21.3 ± 4.3

^1^Female Genital Self-Image Scale

^2^Pelvic floor dysfunction (urinary incontinence and/or pelvic organ prolapse and/or fecal incontinence)

The participants’ FISI mean score was 2.1 ± 6.3, much lower than 38.6 ± 10.7 among uncut women with symptoms of FI and 23.2 ± 15.0 among the general population in the Netherlands (Additional file 1: Table 3) [34]. The participants often complained of constipation during recruitment and interviews, yet the WCS mean score was of 7.4 ± 5.9. The cut-off being set at 15 [28], only 13 women (10.5%) showed pathological scores, with a mean of 18.5 ± 2.8 (Additional file 1: Table 3).

We compared the scores and sub-scores of women with FGM/C type III and those of women with FGM/C types I and II (Additional file 1: Table 4). We also compared non-infibulated (types I, II, III previously defibulated) and infibulated (type III with no previous defibulation or with previous defibulation and reinfibulation) women (Additional file 1: Table 5). The PFDI-20 and PFIQ-7 mean scores and sub-scores were higher among women with FGM/C type III as well as among still infibulated women; however, the differences were not statistically significant. The PISQ-IR, FISI, WCS and FGSIS mean scores were similar in the two groups compared in each case.

We analysed the PFDI-20, UDI-6 and PFIQ-7 scores as a function of variables of interest through a univariate model (Table 5). Women who reported past violent events other than FGM/C and arranged or forced marriage had significantly higher scores in all three scales. Women who were more than 10 at the time of FGM/C, women who had spent less than 6 months in Switzerland, and nulliparous women had significantly higher PFIQ-7 scores. Women older than 35 and overweight women had significantly higher PFDI-20 and UDI-6 scores. Pregnant women had significantly higher UDI-6 scores.

**Table 5:** Univariate models assessing association between selected variables and PFDI-20^1^, UDI-6^2^, PFIQ-7^3^ scores

Variables	PFDI-20^1^		UDI-6^2^		PFIQ-7^2^	
	Mean ± SD (Me: p25-p75)	p-value	Mean ± SD (Me: p25-p75)	p-value	Mean ± SD (Me: p25-p75)	p-value
Type of FGM/C		0.282		0.323		0.136
III	53.7 ± 57.0 (26.1: 12.5–84.6)		21.0 ± 25.3 (10.4: 0.0–33.4)		46.6 ± 56.5 (21.5: 0.0–90.5)	
I, II	43.4 ± 43.4 (28.2: 10.5–68.7)		17.0 ± 20.4 (12.5: 0.0–20.8)		32.0 ± 48.2 (9.5: 0.0–47.6)	
Type of FGM/C		0.067		0.115		0.213
Infibulated	63.4 ± 68.2 (26.1: 13.5–84.6)		25.0 ± 27.4 (12.5: 3.1–50.0)		50.5 ± 62.2 (9.5: 0.0–90.5)	
Non-infibulated	44.3 ± 43.7 (26.6: 20.5–75)		17.5 ± 21.6 (10.4: 0.0–25.0)		37.0 ± 49.9 (19.0: 0.0–57.1)	
Age at FGM/C		0.516		0.247		0.019
< 1 year old	38.5 ± 40.0 (25.0: 7.3–68.7)		14.5 ± 19.7 (4.2: 0.0–20.8)		22.7 ± 34.4 (4.8: 0.0–28.6)	
1–5 years old	61.7 ± 65.0 (30.8: 13.6–103.7)		22.2 ± 27.0 (12.5: 2.1–31.3)		33.3 ± 42.2 (21.5: 7.1–31.0)	
6–10 years old	50.5 ± 52.1 (36.5: 13.0–76.1)		19.7 ± 23.7 (8.3: 0.0–31.3)		46.7 ± 56.9 (19.0: 0.0–81.0)	
> 10 years old	81.6 ± 72.7 (65.6: 10.4–170.8)		32.2 ± 30.7 (16.6: 4.2–66.6)		80.6 ± 73.7 (90.5: 9.5–128.6)	
Unknown/no answer	40.5 ± 41.7 (18.8: 14.6–66.7)		19.1 ± 21.2 (12.5: 4.2–20.8)		44.1 ± 59.7 (19.1: 0.0–57.2)	
Past violent events		0.001		0.002		0.001
Yes	66.9 ± 57.3 (52.1: 17.7–107.3)		26.6 ± 25.7 (16.6: 4.2–52.1)		58.4 ± 59.3 (48.4: 2.4–100.0)	
None/no answer	35.2 ± 42.6 (17.2: 6.8–54.7)		13.8 ± 19.8 (4.2: 0.0–16.7)		26.2 ± 43.7 (4.8: 0.0–31.0)	
Time in Switzerland		0.346		0.835		0.010
< 6 months	79.3 ± 71.1 (73.2: 18.8–87.5)		25.8 ± 27.3 (16.7: 4.2–41.7)		89.5 ± 65.8 (95.3: 23.8–133.3)	
6–12 months	46.1 ± 51.4 (28.7: 13.5–67.8)		16.2 ± 19.6 (8.3: 4.2–20.8)		36.0 ± 51.1 (7.2: 0.0–80.9)	
12–60 months	44.1 ± 45.8 (22.9: 14.6–68.7)		19.2 ± 23.8 (12.5: 0.0–29.2)		42.9 ± 61.7 (9.5: 0.0–57.1)	
> 5 years	50.2 ± 53.3 (25.0: 8.3–79.1)		19.9 ± 24.0 (8.3: 0.0–33.3)		29.9 ± 35.5 (19.0: 0.0–52.4)	
Age in categories		0.001		0.004		0.999
< 25	36.3 ± 31.5 (26.1: 4.2–66.7)		13.9 ± 19.9 (4.2: 0.0–16.6)		37.5 ± 52.6 (0.0: 0.0–81.0)	
25–35	40.4 ± 42.9 (18.8: 12.5–67.8)		15.9 ± 20.4 (8.3: 0.0–20.8)		43.0 ± 57.3 (14.3: 0.0–81.0)	
≥ 35	76.5 ± 68.6 (67.7: 12.5–129.2)		30.5 ± 27.9 (22.9: 4.2–58.3)		38.5 ± 47.8 (23.8: 0.0–57.2)	
Dichotomized BMI		0.006		0.011		0.390
< 25.0	39.4 ± 40.3 (23.0: 9.4–66.2)		15.3 ± 20.1 (8.3: 0.0–18.8)		37.4 ± 49.3 (11.9: 0.0–57.2)	
≥ 25.0	65.5 ± 63.8 (39.1: 13.5–108.2)		26.2 ± 26.8 (16.7: 2.1–50.0)		45.9 ± 60.0 (19.1: 0.0–81.0)	
Ongoing pregnancy		0.153		0.044		0.085
No	54.4 ± 55.7 (35.5: 13.5–77.1)		22.6 ± 25.5 (12.5: 0.0–34.4)		46.8 ± 57.3 (23.8: 0.0–81)	
Yes	40.3 ± 43.3 (17.7: 8.3–70.8)		13.8 ± 17.9 (8.3: 0.0–20.8)		29.3 ± 44.3 (9.5: 0.0–47.6)	
Parity		0.575		0.628		0.010
Nulliparous	53.3 ± 58.4 (32.4: 14.6–69.3)		20.5 ± 25.0 (10.4: 0.0–25.0)		57.6 ± 56.1 (42.9: 0.0–90.5)	
Primiparous	47.5 ± 42.5 (26.1: 12.5–75.0)		20.4 ± 20.4 (12.5: 4.2–29.2)		33.5 ± 55.7 (4.7: 0.0–47.6)	
Multiparous	47.2 ± 51.9 (18.8: 8.3–84.6)		18.1 ± 24.2 (4.2: 0.0–33.3)		29.0 ± 46.1 (9.5: 0.0–44.4)	
Previous episiotomy		0.464		0.231		0.521
No	47.8 ± 53.0 (26.0: 10.4–70.8)		18.2 ± 22.7 (8.3: 0.0–25.0)		43.1 ± 55.0 (19.0: 0.0–81.0)	
Yes	51.1 ± 49.3 (26.1: 12.5–94.8)		21.2 ± 24.0 (12.5: 0.0–33.3)		35.8 ± 51.6 (9.5: 0.0–57.2)	
Unknown	88.0 ± 81.7 (88.0: 30.2–145.8)		43.8 ± 44.2 (43.8: 12.5–75.0)		39.6 ± 56.0 (39.6: 0.0–79.2)	

p-values from linear regression models (univariate analyses)

Other tested variables not shown in the table: previous reinfibulation, origin, smoking, coffee consumption

^1^Pelvic Floor Distress Inventory

^2^Urinary Distress Inventory

^3^Pelvic Floor Impact Questionnaire

We assessed the association between the variables of interest and the PFDI-20, UDI-6 and PFIQ-7 scores through a multivariable model (Table 6). Past violent events were significantly associated with higher PFDI-20 and PFIQ-7 scores, whereas a period of staying in Switzerland of less than 6 months and nulliparity were significantly associated with higher PFIQ-7 scores, independently of age, weight, ongoing pregnancy and history of episiotomy. Although age at FGM/C as a variable was not significantly associated with higher scores for pelvic floor impact and distress, age at FGM/C of more than 10 was significantly associated with higher PFIQ-7 scores when compared with age at FGM/C of less than 1. Age at FGM/C and type of FGM/C were collinear—most types III were performed at more than 6, most types I and II at less than 1 (data not shown)—but the addition of one did not change the association between the other and the outcome scores.

**Table 6:** Multivariable model assessing association between selected variables and PFDI-20^1^, UDI-6^2^ PFIQ-7^3^ scores

Variables	PFDI-20^1^		UDI-6^2^		PFIQ-7^3^	
	Mean ± SD (95% CI)	p-value	Mean ± SD (95% CI)	p-value	Mean ± SD (95% CI)	p-value
Type of FGM/C						
III	49.4 ± 8.4 (37.8 to 61.1)	–	19.8 ± 2.7 (14.3 to 25.2)	–	43.6 ± 5.8 (32.2 to 55.1)	–
I, II	49.6 ± 6.8 (35.1 to 64.1)	0.985	19.2 ± 3.4 (12.4 to 26.0)	0.901	36.4 ± 7.2 (22.1 to 50.7)	0.461
Age at FGM/C		0.276		0.589		0.058
< 1 year old	42.2 ± 8.8 (24.8 to 59.6)	–	17.0 ± 4.1 (8.9 to 25.1)	–	28.6 ± 8.7 (11.4 to 45.7)	–
1–5 years old	63.8 ± 12.4 (39.2 to 88.4)	0.170	22.5 ± 5.8 (11.0 to 34.0)	0.456	32.7 ± 12.3 (8.4 to 57.0)	0.791
6–10 years old	46.3 ± 8.0 (30.3 to 62.2)	0.743	17.6 ± 3.8 (10.2 to 25.1)	0.915	41.2 ± 7.9 (25.5 to 56.9)	0.309
> 10 years old	75.3 ± 15.1 (45.3 to 105.2)	0.063	29.3 ± 7.1 (15.3 to 43.3)	0.140	79.2 ± 14.9 (49.7 to 108.8)	0.004
Unknown/no answer	43.8 ± 10.8 (22.4 to 65.1)	0.908	20.1 ± 5.0 (10.1 to 30.1)	0.628	45.8 ± 10.6 (24.7 to 66.9)	0.207
Past violent events						
Yes	61.4 ± 6.7 (48.2 to 74.7)	–	24.2 ± 3.1 (18.0 to 30.3)	–	55.1 ± 6.6 (42.0 to 68.1)	–
None/no answer	39.7 ± 6.0 (27.8 to 51.6)	0.021	15.8 ± 2.8 (10.2 to 21.3)	0.057	28.9 ± 6.0 (17.1 to 40.7)	0.005
Time in Switzerland		0.108		0.507		0.013
< 6 months	84.5 ± 16.0 (52.9 to 116.1)	-	28.8 ± 7.5 (14.0 to 43.6)	–	85.0 ± 15.7 (53.8 to 116.2)	–
6–12 months	56.7 ± 11.9 (33.0 to 80.3)	0.166	20.0 ± 5.6 (9.0 to 31.0)	0.347	40.5 ± 11.8 (17.2 to 63.9)	0.026
12–60 months	46.9 ± 7.0 (33.0 to 60.8)	0.034	20.4 ± 3.3 (13.9 to 26.9)	0.307	44.5 ± 7.0 (30.7 to 58.2)	0.021
> 5 years	42.0 ± 7.3 (27.5 to 56.6)	0.018	16.5 ± 3.4 (9.8 to 23.3)	0.140	27.5 ± 7.2 (13.2 to 41.8)	0.001
Age in categories		0.035		0.140		0.283
< 25	38.6 ± 11.1 (16.5 to 60.7)	–	14.7 ± 5.2 (4.4 to 25.0)	–	31.6 ± 11.0 (9.8 to 53.4)	–
25–35	42.5 ± 6.1 (30.4 to 54.7)	0.762	17.5 ± 2.9 (11.8 to 23.2)	0.652	47.6 ± 6.1 (35.6 to 59.6)	0.214
≥ 35	70.6 ± 9.1 (52.5 to 88.7)	0.036	27.0 ± 4.3 (18.5 to 35.4)	0.085	33.4 ± 9.0 (15.5 to 51.2)	0.906
Dichotomized BMI						
< 25.0	44.9 ± 5.9 (33.2 to 56.7)	–	17.6 ± 2.8 (12.1 to 23.0)	–	38.7 ± 5.8 (27.1 to 50.3)	–
≥ 25.0	56.7 ± 7.7 (41.4 to 72.1)	0.265	22.7 ± 3.6 (15.6 to 29.9)	0.293	44.0 ± 7.6 (28.8 to 59.1)	0.609
Ongoing pregnancy						
Yes	46.0 ± 7.9 (30.4 to 61.6)	–	21.5 ± 2.6 (16.3 to 26.6)	–	32.5 ± 7.8 (17.1 to 47.9)	–
No	51.4 ± 5.5 (40.4 to 62.4)	0.595	15.9 ± 3.7 (8.6 to 23.2)	0.240	45.1 ± 5.5 (34.3 to 56.0)	0.206
Parity		0.609		0.452		0.021
Nulliparous	55.4 ± 8.5 (38.6 to 72.3)	–	22.5 ± 4.0 (14.7 to 30.4)	–	60.5 ± 8.4 (43.9 to 77.1)	–
Primiparous	51.0 ± 9.1 (33.0 to 69.0)	0.729	21.3 ± 4.2 (12.9 to 29.7)	0.841	36.6 ± 8.9 (18.9 to 54.3)	0.062
Multiparous	42.7 ± 8.1 (26.6 to 58.1)	0.335	15.5 ± 3.8 (8.0 to 23.0)	0.255	24.1 ± 8.0 (8.2 to 40.0)	0.006
Previous episiotomy		0.584		0.412		0.348
Yes	57.8 ± 9.0 (39.8 to 75.7)	–	23.6 ± 4.2 (15.3 to 32.0)	–	51.9 ± 8.9 (34.2 to 69.6)	–
No	45.5 ± 5.8 (34.1 to 57.0)	0.301	17.3 ± 2.7 (12.0 to 22.7)	0.251	35.2 ± 5.7 (23.9 to 46.5)	0.151
Unknown	52.9 ± 37.2 (− 20.8 to 126.7)	0.899	32.8 ± 17.4 (− 1.6 to 67.3)	0.385	52.6 ± 36.7 (− 20.2 to 125.4)	0.644

p-values from linear regression models (multivariable analysis)

Adjusted R^2^ of the model = 0.1513 (PFDI-20), 010,830 (UDI-6), 0.2134 (PFIQ-7)

Residuals were reasonably normally distributed (visual inspection)

^1^Pelvic Floor Distress Inventory

^2^Urinary Distress Inventory

^3^Pelvic Floor Impact Questionnaire

## Discussion

Our sample of 124 women from different ethnic origins, with different types of FGM/C, and without POP, presented with: scores for pelvic floor symptoms’ distress and impact that were higher than those of uncut women without PFD and lower than those of uncut women with PFD [21, 32, 33]; scores for sexual function that were similar to those of uncut women with PFD [23]; scores for faecal incontinence and constipation that were lower than those of uncut women with or without PFD [28, 34]; scores that showed a satisfactory genital self-image [30].

FGM/C, particularly type III, can be associated with urogynaecological complications due to injuries of the genitourinary tracts, scarring process, and physiologic changes such as obstructed micturition after infibulation, potentially leading to overactive bladder and urge incontinence [10, 13, 14, 35]. As expected, we found higher scores for urogynaecological symptoms’ distress and impact in infibulated women. However, the differences were not significant. Our sample size was not calculated for comparing different types of FGM/C and might lack the power to show a statistical difference. Nulliparity was significantly associated with higher scores for distress and impact of pelvic floor symptoms. This might seem counterintuitive, as parity is commonly associated with PFD [36]. However, many participants, especially with FGM/C type III, reported genitourinary or sexual symptoms decreased or resolved after their first vaginal birth, which was the moment when defibulation (surgical opening of the infibulation [35]) or perineal tear happened, widening the vaginal introitus and/or uncovering the urethral meatus.

Psychiatric comorbidities and history of trauma or abuse are risk factors for greater severity of urogynaecological symptoms [37]. It has been shown that FGM/C can cause psychosexual disorders such as dyspareunia, vaginismus, anxiety and PTSD [1, 15] and is often associated with history of other violence and *polyvictimisation* [38–40]. We found that almost one in two participants reported past violent events other than FGM/C or forced/arranged marriage, and that past violent events were significantly associated with higher scores of distress and impact of pelvic floor symptoms. It has also been shown that vivid recollection of the cutting increases the risk of mental health conditions such as PTSD, depression and anxiety [41]. We found that all participants who underwent FGM/C at age more than 10 clearly remembered their cutting, in contrast to those who had it during early childhood or infancy, and that age at FGM/C of more than 10 was significantly associated with greater impact of pelvic floor symptoms when compared with age at FGM/C of less than 1, with a collinearity between older age at FGM/C and a more severe FGM/C (type III).

A longer period of staying in Switzerland could have been thought to be associated with higher urogynaecological symptoms’ severity and impact due to increased awareness of symptoms or to a feeling of being different than uncut women. However, we found the opposite. Since most of the participants were recruited at the FGM/C outpatient clinic where they had access to medical and psychological care, newer arrivals might have received fewer treatments and experience more symptoms.

Our study sample showed a positive genital self-image and a satisfactory sexual function and quality. Possible positive sociocultural beliefs associated with FGM/C [5, 6] could explain the positive genital self-image and thus contribute to a good sexual response, even in presence of pelvic floor symptoms. Our clinical experience as well as previous literature have shown that even though women with different types of FGM/C and complications can significantly suffer from dyspareunia and sexual dysfunction, they can also report a satisfactory sexual life, sexual pleasure and orgasm. First, the clitoris is not involved by all types of FGM/C and when cut, most of the tumescent structures responsible for the sexual response are not removed. Second, some “compensatory” psychophysical and cultural mechanisms have been suggested to be able to overcome the “anatomical damage”, such as the enhancement of stimuli originating from other body areas, or through positive emotions, cultural values and fantasies [42–45]. Non-sexually active participants mostly attributed their sexual inactivity to the absence of a partner and not to a health condition, yet their scores suggest that their sexual inactivity and poor sexual quality might also be linked to a medical condition.

The strengths of our study are the inclusion of women with different types of FGM/C and from different origins and backgrounds, the systematic gynaecological examination prior the administration of questionnaires, and the use of validated scoring systems. A limitation of our study was that the questionnaires were not validated in women with FGM/C, and therefore did not capture information specific to the cutting and cultural issues. Nevertheless, the participants showed good understanding and acceptance of the questions and showed great interest in the study in order to raise awareness among healthcare professionals, and no one interrupted the interview. Another limitation might be that one third of the participants was pregnant at the time of the interview, half during the third trimester. Pregnancy might have worsened pelvic floor symptoms [46], indeed pregnant participants had significantly higher UDI-6 mean scores.

The presence of interpreters might have caused the loss of some information or embarrassment during the interviews. However, we consider a strength avoiding the bias of selecting women who could speak French or English because of having lived in the West for a longer time or because of higher economic or education backgrounds. We also acknowledge that an oral translation provided by multiple interpreters, even if trained and certified, is less expensive but also less consistent than a written translation of the questionnaires in the different original languages of the participants.

## Conclusions

In conclusion, women with FGM/C, without POP, can suffer from pelvic floor symptoms responsible for distress and impact on their daily life, due to both anatomical and psychological factors. Healthcare professionals should be able to screen, recognize, record and treat urogynaecological and psychosexual disorders in women and girls living with FGM/C, even when symptoms are not spontaneously disclosed or recognized by the client.

Surgical defibulation, clitoral reconstruction and treatment of scar complications, obstetric trauma repair, pelvic floor therapy, counselling and psychosexual care are existing treatments [47, 48]. Care should begin from starting the conversation and giving culturally sensitive information, with certified interpreters when needed.

Future research could include a control group and focus on possible prevention strategies and treatments.

## Supplementary Information

**Additional file 1: Table 1.** Complementary sociodemographic information. **Table 2.** Complementary background information. **Table 3.** FISI^1^ and WCS^2^ mean scores of our sample and from the literature [28, 34]. **Table 4.** Comparison between women with FGM/C type III and those with FGM/C types I and II.** Table 5.** Comparison between still infibulated and non-infibulated women.

## Data Availability

The datasets used and analysed during the current study are available from the corresponding author on reasonable request.

## Abbreviations

FGM/C: Female Genital Mutilation/Cutting
BMI: Body Mass Index
POP: Pelvic organ prolapse
PTSD: Post-traumatic stress disorder
PFD: Pelvic floor dysfunction
PFDI-20: Pelvic Floor Distress Inventory short-form
UDI-6: Urinary Distress Inventory
POPDI-8: Pelvic Organ Prolapse Distress Inventory
CRADI-6: ColoRectal-Anal Distress Inventory
PFIQ-7: Pelvic Floor Impact Questionnaire short-form
UIQ-7: Urinary Impact Questionnaire
POPIQ-7: Pelvic Organ Prolapse Impact Questionnaire
CRAIQ-7: ColoRectal-Anal Impact Questionnaire
PISQ-IR: Pelvic organ prolapse Incontinence Sexual Questionnaire—IUGA Revised
IUGA: International UroGynaecological Association
SA: Sexually active
NSA: Non-sexually active
AO: Arousal and orgasm
PR: Partner-related impact on (in)activity
CS: Condition-specific impact on (in)activity
CI: Condition-specific Impact on quality
D: Desire
GQ: Global quality
FISI: Fecal Incontinence Severity Index
WCS: Wexner’s constipation scoring system
FGSIS: Female Genital Self-Image Scale
SD: Standard deviation
CI: Confidence interval

## References

[1] World Health Organization. Female genital mutilation fact sheet. 2020; http://www.who.int/en/news-room/fact-sheets/detail/female-genital-mutilation.

[2] World Health Organization. Types of female genital mutilation. 2020; https://www.who.int/teams/sexual-and-reproductive-health-and-research/areas-of-work/female-genital-mutilation/types-of-female-genital-mutilation.

[3] World Health Organization. Prevanence of female genital mutilation. 2020; http://www.who.int/reproductivehealth/topics/fgm/prevalence/en/.

[4] United Nations Population Fund. Female Genital Mutilation dashboard. https://www.unfpa.org/fr/data/dashboard/fgm.

[5] Freymeyer RH, Johnson BE (2007). An exploration of attitudes toward Female Genital Cutting in Nigeria. Popul Res Policy Rev.

[6] Berggren V, Abdel Salam G, Bergstrom S, Johansson E, Edberg AK (2004). An explorative study of Sudanese midwives' motives, perceptions and experiences of re-infibulation after birth. Midwifery.

[7] Van Baelen L, Ortensi L, Leye E (2016). Estimates of first-generation women and girls with Female Genital Mutilation in the European Union, Norway and Switzerland. Eur J Contracept Reprod Health Care.

[8] Goldberg H, Stupp P, Okoroh E, Besera G, Goodman D, Danel I (2016). Female Genital Mutilation/Cutting in the United States: updated estimates of women and girls at risk, 2012. Public Health Rep.

[9] Fedpol. Le Conseil fédéral renforce les mesures contre les Mutilations Génitales Féminines. 2020; https://www.fedpol.admin.ch/fedpol/fr/home/aktuell/mm.msg-id-81313.html.

[10] Nour NM (2004). Female Genital Cutting: clinical and cultural guidelines. Obstet Gynecol Surv.

[11] World Health Organization. Health risks of female genital mutilation. 2020; https://www.who.int/teams/sexual-and-reproductive-health-and-research/areas-of-work/female-genital-mutilation/health-risks-of-female-genital-mutilation.

[12] Abdulcadir J, Rodriguez MI, Say L (2015). Research gaps in the care of women with female genital mutilation: an analysis. BJOG.

[13] Teufel K, Dorfler DM (2013). Female Genital Circumcision/Mutilation: implications for female urogynaecological health. Int Urogynecol J.

[14] Amin MM, Rasheed S, Salem E (2013). Lower urinary tract symptoms following female genital mutilation. Int J Gynaecol Obstet.

[15] McCool-Myers M, Theurich M, Zuelke A, Knuettel H, Apfelbacher C (2018). Predictors of female sexual dysfunction: a systematic review and qualitative analysis through gender inequality paradigms. BMC Womens Health.

[16] Banks E, Meirik O, Farley T, Akande O, Bathija H, Ali M (2006). Female Genital Mutilation and obstetric outcome: WHO collaborative prospective study in six African countries. Lancet (London, England).

[17] Johansen REB, Ziyada MM, Shell-Duncan B, Kaplan AM, Leye E (2018). Health sector involvement in the management of Female Genital Mutilation/Cutting in 30 countries. BMC Health Serv Res.

[18] Vloeberghs E, van der Kwaak A, Knipscheer J, van den Muijsenbergh M (2012). Coping and chronic psychosocial consequences of female genital mutilation in The Netherlands. Ethn Health.

[19] World Health Organization. Care of girls and women living with Female Genital Mutilation: a clinical handbook. 2018; http://apps.who.int/iris/bitstream/handle/10665/272429/9789241513913-eng.pdf.

[20] Toubia N (2000). Caring for women with circumcision: a technical manual for health care providers. BMJ.

[21] Barber MD, Walters MD, Bump RC (2005). Short forms of two condition-specific quality-of-life questionnaires for women with pelvic floor disorders (PFDI-20 and PFIQ-7). Am J Obstet Gynecol.

[22] de Tayrac R, Deval B, Fernandez H, Mares P (2007). Development of a linguistically validated French version of two short-form, condition-specific quality of life questionnaires for women with pelvic floor disorders (PFDI-20 and PFIQ-7). J Gynecol Obstet Biol Reprod.

[23] Rogers RG, Rockwood TH, Constantine ML (2013). A new measure of sexual function in women with Pelvic Floor Disorders (PFD): the Pelvic organ prolapse/Incontinence Sexual Questionnaire, IUGA-Revised (PISQ-IR). Int Urogynecol J.

[24] Rogers RG, Espuna Pons ME (2013). The Pelvic organ prolapse Incontinence Sexual Questionnaire, IUGA-revised (PISQ-IR). Int Urogynecol J.

[25] Rockwood TH, Constantine ML, Adegoke O (2013). The PISQ-IR: considerations in scale scoring and development. Int Urogynecol J.

[26] Fatton B, Hermieu JF, Cour F, Wagner L, Jacquetin B, de Tayrac R (2013). French language validation of the Pelvic Organ Prolapse/Urinary Incontinence Sexual Questionnaire—IUGA revised (PISQ-IR). Prog Urol.

[27] Rockwood TH, Church JM, Fleshman JW (1999). Patient and surgeon ranking of the severity of symptoms associated with Fecal Incontinence: the Fecal Incontinence Severity Index. Dis Colon Rectum.

[28] Agachan F, Chen T, Pfeifer J, Reissman P, Wexner SD (1996). A constipation scoring system to simplify evaluation and management of constipated patients. Dis Colon Rectum.

[29] Herbenick D, Reece M (2010). Development and validation of the Female Genital Self-Image Scale. J Sex Med.

[30] Herbenick D, Schick V, Reece M, Sanders S, Dodge B, Fortenberry JD (2011). The Female Genital Self-Image Scale (FGSIS): results from a nationally representative probability sample of women in the United States. J Sex Med.

[31] Hulley SB, Cummings SR, Browner WS, Grady D, Newman TB. In: Hulley SB, et al. editors. Designing clinical research, 4th ed. Lippincott Williams & Wilkins (LWW); 2013. ISBN: 978-1-60-831804-9.

[32] Henn EW, Richter BW, Marokane MMP (2017). Validation of the PFDI-20 and PFIQ-7 quality of life questionnaires in two African languages. Int Urogynecol J.

[33] Utomo E, Blok BF, Steensma AB, Korfage IJ (2014). Validation of the Pelvic Floor Distress Inventory (PFDI-20) and Pelvic Floor Impact Questionnaire (PFIQ-7) in a Dutch population. Int Urogynecol J.

[34] t Hoen LA, Utomo E, Schouten WR, Blok BF, Korfage IJ (2017). The Fecal Incontinence Quality of Life scale (FIQL) and Fecal Incontinence Severity Index (FISI): validation of the Dutch versions. Neurourol Urodyn.

[35] Abdulcadir J, Dällenbach P (2013). Overactive bladder after female genital mutilation/cutting (FGM/C) type III. BMJ Case Rep.

[36] Wu JM, Vaughan CP, Goode PS (2014). Prevalence and trends of symptomatic Pelvic Floor Disorders in U.S. women. Obstet Gynecol.

[37] Klausner AP, Ibanez D, King AB (2009). The influence of psychiatric comorbidities and sexual trauma on lower urinary tract symptoms in female veterans. J Urol.

[38] Antonetti Ndiaye E, Fall S, Beltran L (2015). Intérêt de la prise en charge pluridisciplinaire des femmes excisées. J Gynécol Obstét Biol Reprod.

[39] Im H, Swan LET, Heaton L (2019). Polyvictimization and mental health consequences of female genital mutilation/circumcision (FGM/C) among Somali refugees in Kenya. Women Health.

[40] Lever H, Ottenheimer D, Teysir J, Singer E, Atkinson HG (2018). Depression, anxiety, post-traumatic stress disorder and a history of pervasive gender-based violence among women asylum seekers who have undergone Female Genital Mutilation/Cutting: a retrospective case review. J Immigr Minor Health.

[41] Knipscheer J, Vloeberghs E, van der Kwaak A, van den Muijsenbergh M (2015). Mental health problems associated with female genital mutilation. BJPsych Bull.

[42] Abdulcadir J, Dewaele R, Firmenich N (2020). In vivo imaging-based 3-dimensional pelvic prototype models to improve education regarding sexual anatomy and physiology. J Sex Med.

[43] Catania L, Abdulcadir O, Puppo V, Verde JB, Abdulcadir J, Abdulcadir D (2007). Pleasure and orgasm in women with Female Genital Mutilation/Cutting (FGM/C). J Sex Med.

[44] Buggio L, Facchin F, Chiappa L, Barbara G, Brambilla M, Vercellini P (2019). Psychosexual consequences of female genital mutilation and the impact of reconstructive surgery: a narrative review. Health Equity.

[45] Berg RC, Denison E (2012). Does Female Genital Mutilation/Cutting (FGM/C) affect women’s sexual functioning? A systematic review of the sexual consequences of FGM/C. Sex Res Soc Policy.

[46] van Brummen HJ, Bruinse HW, van der Bom JG, Heintz AP, van der Vaart CH (2006). How do the prevalences of urogenital symptoms change during pregnancy?. Neurourol Urodyn.

[47] Berg RC, Taraldsen S, Said MA, Sørbye IK, Vangen S (2018). The effectiveness of surgical interventions for women with FGM/C: a systematic review. BJOG.

[48] Abdulcadir J, Catania L (2020). Conceptualizing sexual pain in women with Female Genital Mutilation/Cutting. Arch Sex Behav.

