# Supplementary material for: A cross-sectional study on pelvic floor symptoms in women living with Female Genital Mutilation/Cutting
Source: Reprod Health. 2021 Feb 12;18:39. doi: 10.1186/s12978-021-01097-9 (PMC7881631; doi:10.1186/s12978-021-01097-9)
Supplement: Supplementary file 1 — Additional file 1: Table 1. Complementary sociodemographic information. Table 2. Complementary background information. Table 3. FISI1 and WCS2 mean scores of our sample and from the literature [28, 34]. Table 4. Comparison between women with FGM/C type III and those with FGM/C types I and II. Table 5. Comparison between still infibulated and non-infibulated women. [file 12978_2021_1097_MOESM1_ESM.docx]

# ADDITIONAL FILES

Additional table 1: Complementary sociodemographic information

| Variable | n (%) |
| --- | --- |
| Country of origin  Eritrea  Somalia  Ethiopia  Guinea  Burkina Faso  Other ^1^ | 40 (32.3)  40 (32.3)  9 (7.2)  9 (7.2)  5 (4.1)  21 (16.9) |
| Mother tongue  Somali  Tigrinya  Amharic  Arabic  Other ^2^ | 41 (33.1)  39 (31.5)  7 (5.6)  4 (3.2)  33 (26.6) |
| Language spoken during the study  French  English  Certified interpreter | 56 (45.2)  20 (16.1)  48 (38.7) |
| Religion  Muslim  Christian  Atheist | 74 (59.7)  49 (39.5)  1 (0.8) |
| Marital status  Single  Married / in a relationship  Divorced / separated | 18 (14.5)  89 (71.8)  17 (13.7) |
| Arranged/forced marriage (n_tot_ = 100)  Yes  No  No answer | 26 (26.0) (18 married, 8 divorced)  70 (70.0) (2 single, 60 married, 8 divorced)  4 (4.0) (1 single, 2 married, 1 divorced) |
| Education  None  Primary  Secondary  Other ^3^ | 17 (13.7)  69 (55.7)  33 (26.6)  5 (4.0) |
| Profession  No / housewife  Student  Technical/unqualified position  Intellectual position  Disability insurance  No Answer | 77 (62.1)  8 (6.4)  26 (21.0)  10 (8.1)  2 (1.6)  1 (0.8) |
| Smoking  Yes  No | 2 (1.6)  122 (98.4) |
| Coffee consumption  Yes  No | 57 (46.0)  67 (54.0) |

^1^ Other ethnic origin: Burkina Faso, Chad, Djibouti, Gambia, Guinea, Guinea-Bissau, Ivory Coast, Mali, Nigeria, Senegal, Sierra Leone, Sudan, Togo
^2^ Other mother tongues: French, English, African languages
^3^ Other education statuses: integration classes, internships, language courses, home schooling

Additional table 2: Complementary background information

| Variable | n (%) |
| --- | --- |
| Setting of FGM/C  At home, by a traditional exciser  At home, by a healthcare provider  At home, unknown by whom  At the hospital or in a medical setting  Unknown | 72 (58.5)  15 (12.2)  3 (2.5)  7 (5.7)  26 (21.1) |
| Acute complications of FGM/C ^1^  Yes  No  Unknown | 50 (40.3)  27 (21.8)  47 (37.9) |
| Chronic complications of FGM/C ^2^  Yes  No  Unknown | 60 (48.4)  48 (38.7)  16 (12.9) |
| Gravidity (number of pregnancies)  Nulligravida  Primigravida  Multigravida | 2.1 ± 1.8 (min=0, max=8)  24 (19.3)  28 (22.6)  72 (58.1) |
| Parity (number of childbirths)  Nulliparous  Primiparous  Multiparous | 1.3 ± 1.4 (min=0, max=6)  46 (37.1)  31 (25.0)  47 (37.9) |
| Assisted delivery: vacuum, forceps (n_tot_ = 78)  No  Yes  Unknown | 64 (82.1)  13 (16.7)  1 (1.2) |
| Mediolateral episiotomy (n_tot_ = 78)  No  Yes  Unknown | 37 (47.4)  39 (50.0)  2 (2.6) |
| Perineal tear (n_tot_ = 78)  No  1^st^ or 2^nd^ degree  3^rd^ or 4^th^ degree  Unknown | 42 (53.8)  34 (43.6)  0 (0.0)  2 (2.6) |
| Other gynaecological operations ^3^  Yes, linked to FGM/C  Yes, not linked to FGM/C  No  Unknown | 3 (2.4)  30 (24.2)  89 (71.8)  2 (1.6) |

^1^ Acute complications: hemorrhage, pain, urinary pain, infection
^2^ Chronic complications: menstrual, sexual, urinary, scarring, obstetric
^3^ Surgical history: dilatation-curettage, myomectomy, hysteroscopic polypectomy, ovarian cystectomy, salpingectomy

Additional table 3: FISI ^1^ and WCS ^2^ mean scores of our sample and from the literature [^28^](#_ENREF_28)^,^[^34^](#_ENREF_34)

|  | Mean scores ± SD of women with FGM/C | | | | Mean scores ± SD of women without FGM/C | |
| --- | --- | --- | --- | --- | --- | --- |
|  | All (n=124) | I (n=9) | II (n=41) | III (n=74) | Without PFD ^3^ | With PFD ^3^ |
| FISI ^1^ | 2.1 ± 6.3 (n=122) | 0.0 ± 0.0 (n=9) | 1.0 ± 4.0 (n=41) | 3.0 ± 8.0 (n=71) | 23.2 ± 15.0 | 38.6 ± 10.7 |
| WCS ^2^ | 7.4 ± 5.9 (n=122) | 6.0 ± 5.0 (n=9) | 7.0 ± 6.0 (n=41) | 8.0 ± 6.0 (n=72) |  | > 15 |

^1^ Fecal Incontinence Severity Index; ^2^ Wexner Constipation Scale; ^3^ Pelvic Floor Dysfunction (Urinary Incontinence and/or Pelvic Organ Prolapse and/or Fecal Incontinence)

Additional table 4: Comparison between women with FGM/C type III and those with FGM/C types I and II

|  | Types of FGM/C | |  | p-values |
| --- | --- | --- | --- | --- |
|  | III (n=74) | I, II (n=50) |  |  |
| PFDI-20 ^1^  UDI-6 ^2^  POPDI-6 ^3^  CRADI-8 ^4^ | 53.7 ± 57.0  21.3± 25.3  13.7 ± 20.4  18.6 ± 19.3 | 43.4 ± 43.4  17.0 ± 20.4  10.0 ± 16.1  16.5 ± 18.5 |  | 0.6174  0.7272  0.3689  0.5961 |
| PFIQ-7 ^5^  UIQ-7 ^6^  POPIQ-7 ^7^  CRAIQ-7 ^8^ | 46.6 ± 56.5  10.0 ± 18.6  20.0 ± 32.7  9.4 ± 20.6 | 32.0 ± 48.3  6.7 ± 18.1  11.7 ± 23.1  7.7 ± 18.5 |  | 0.3242  0.3252  0.5041  0.8218 |
| PISQ-IR ^9^  SA-AO ^9^  SA-PR ^9^  SA-CS ^9^  SA-CI ^9^  SA-D ^9^  SA-GQ ^9^  NSA-CS ^9^  NSA-PR ^9^  NSA-CI ^9^  NSA-GQ ^9^ | 3-3 ± 1.2 (n=50)  3.4 ± 0.7 (n=49)  4.5 ± 0.7 (n=49)  3.8 ± 0.5 (n=49)  2.9 ± 1.0 (n=49)  4.1 ± 1.0 (n=46)  1.5 ± 0.8 (n=23)  2.6 ± 0.8 (n=23)  1.3 ± 0.7 (n=23)  2.6 ± 1.2 (n=23) | 3.4 ± 0.9 (n=35)  3.5 ± 0.6 (n=35)  4.4 ± 1.0 (n=35)  3.8 ± 0.5 (n=34)  3.0 ± 1.0 (n=34)  3.6 ± 1.2 (n=34)  1.3 ± 0.5 (n=14)  2.6 ± 0.9 (n=12)  1.1 ± 0.3 (n=14)  1.9 ± 1.1 (n=14) |  | 0.7101  0.4036  0.8998  0.4395  0.9074  0.1091  0.7124  0.7139  0.2205  0.0924 |
| FISI ^10^  Gas  Mucus  Liquid stool  Solid stool | 2.8 ± 7.5 (n=72)  5.4 ± 1.3 (n=72)  6.0 ± 0.4 (n=72)  5.8 ± 0.8 (n=72)  5.9 ± 0.3 (n=72) | 1.1 ± 3.9  5.7 ± 0.9  6.0 ± 0.3  6.0 ± 0.1  6.0 ± 0.0 |  | 0.2025  0.2163  0.7784  0.3172  0.2366 |
| WCS ^11^ | 7.7 ± 6.1 (n=72) | 6.9 ± 5.4 |  | 0.4876 |
| FGSIS ^12^ | 21.4 ± 5.5 (n=68) | 20.2 ± 6.0 |  | 0.3037 |

^1^ Pelvic Floor Distress Inventory; ^2^ Urinary Distress Inventory; ^3^ Pelvic Organ Prolapse Distress Inventory; ^4^ ColoRectal-Anal Distress Inventory;
^5^ Pelvic Floor Impact Questionnaire; ^6^ Urinary Impact Questionnaire; ^7^ Pelvic Organ Prolapse Impact Questionnaire; ^8^ ColoRectal-Anal Impact Questionnaire;
^9^ Pelvic organ prolapse Incontinence Sexual Questionnaire - IUGA Revised (PISQ-IR) for Sexually Active (SA) and Non-Sexually Active (NSA), Arousal and Orgasm (AO), Partner-Related impact on activity/inactivity (PR), Condition-Specific impact on activity/inactivity (CS), Condition-specific Impact on quality (CI), Desire (D), and Global Quality (GQ);
^10^ Fecal Incontinence Severity Scale; ^11^ Wexner Constipation Score; ^12^ Female Genital Self-Image Scale
^13^ FGM/C type III infibulated and defibulated
p-values from Kruskal-Wallis tests

Additional table 5: Comparison between still infibulated and non-infibulated women

|  | Types of FGM/C | | p-values |
| --- | --- | --- | --- |
|  | Infibulated (n=14) | Non-infibulated (n=90) |  |
| PFDI-20 ^1^  UDI-6 ^2^  POPDI-6 ^3^  CRADI-8 ^4^ | 63.4 ± 68.2  25.0 ± 27.4  18.0 ± 25.2  20.4 ± 22.4 | 44.3 ± 43.7  17.5 ± 21.6  10.0 ± 15.4  16.8 ± 17.5 | 0.3624  0.2218  0.2815  0.6503 |
| PFIQ-7 ^5^  UIQ-7 ^6^  POPIQ-7 ^7^  CRAIQ-7 ^8^ | 50.5 ± 62.2  12.1 ± 23.5  21.4 ± 34.7  10.8 ± 24.1 | 37.0 ± 49.9  7.4 ± 16.0  14.8 ± 27.2  7.9 ± 17.8 | 0.7958  0.4563  0.9150  0.9706 |
| PISQ-IR ^9^  SA-AO ^9^  SA-PR ^9^  SA-CS ^9^  SA-CI ^9^  SA-D ^9^  SA-GQ ^9^  NSA-CS ^9^  NSA-PR ^9^  NSA-CI ^9^  NSA-GQ ^9^ | 3-3 ± 1.3 (n=22)  3.3 ± 0.7 (n=22)  4.4 ± 0.8 (n=21)  3.7 ± 0.6 (n=21)  2.5 ± 1.0 (n=21)  3.8 ± 1.2 (n=19)  1.5 ± 0.8 (n=12)  3.0 ± 0.9 (n=12)  1.4 ± 0.9 (n=12)  2.6 ± 1.1 (n=12) | 3.4 ± 0.9 (n=63)  3.5 ± 0.6 (n=62)  4.5 ± 0.9 (n=63)  3.8 ± 0.4 (n=61)  3.1 ± 1.0 (n=62)  3.9 ± 1.1 (n=61)  1.5 ± 0.7 (n=25)  2.5 ± 0.8 (n=23)  1.2 ± 0.4 (n=24)  2.2 ± 1.2 (n=25) | 0.2430  0.2291  0.2639  0.6825  0.0555  0.5260  0.9391  0.0724  0.8361  0.2851 |
| FISI ^10^  Gas  Mucus  Liquid stool  Solid stool | 2.0 ± 7.0 (n=31)  5.0 ± 1.0 (n=32)  6.0 ± 1.0 (n=32)  6.0 ± 1.0 (n=32)  6.0 ± 0.0 (n=32) | 2.1 ± 6.1  5.6 ± 1.1  6.0 ± 0.3  6.0 ± 0.5  6.0 ± 0.3 | 0.6328  0.6176  0.7613  0.7733  0.3971 |
| WCS ^11^ | 8.0 ± 7.0 (n=32) | 7.0 ± 5.4 | 0.3689 |
| FGSIS ^12^ | 20.0 ± 6.0 (n=31) | 21.1 ± 5.6 | 0.5588 |

^1^ Pelvic Floor Distress Inventory; ^2^ Urinary Distress Inventory; ^3^ Pelvic Organ Prolapse Distress Inventory; ^4^ ColoRectal-Anal Distress Inventory;
^5^ Pelvic Floor Impact Questionnaire; ^6^ Urinary Impact Questionnaire; ^7^ Pelvic Organ Prolapse Impact Questionnaire; ^8^ ColoRectal-Anal Impact Questionnaire;
^9^ Pelvic organ prolapse Incontinence Sexual Questionnaire - IUGA Revised (PISQ-IR) for Sexually Active (SA) and Non-Sexually Active (NSA), Arousal and Orgasm (AO), Partner-Related impact on activity/inactivity (PR), Condition-Specific impact on activity/inactivity (CS), Condition-specific Impact on quality (CI), Desire (D), and Global Quality (GQ);
^10^ Fecal Incontinence Severity Scale; ^11^ Wexner Constipation Score; ^12^ Female Genital Self-Image Scale
^13^ FGM/C type III infibulated and defibulated; ^14^ FGM/C types I, II, III defibulated
p-values from Kruskal-Wallis tests
